# Tobacco price and use following California Proposition 56 tobacco tax increase

**DOI:** 10.1371/journal.pone.0257553

**Published:** 2021-10-13

**Authors:** Christian Gunadi, Tarik Benmarhnia, Martha White, John P. Pierce, Sara B. McMenamin, Eric C. Leas, Yuyan Shi

**Affiliations:** 1 Herbert Wertheim School of Public Health and Human Longevity Science, University of California San Diego, La Jolla, California, United States of America; 2 Scripps Institution of Oceanography, University of California San Diego, La Jolla, California, United States of America; Medical University of South Carolina, UNITED STATES

## Abstract

**Background:**

California Proposition 56 increased cigarette excise tax by $2 per pack with equivalent increases on non-cigarette tobacco products. We estimated the changes in cigarette price, cigarette use, and non-cigarette use following the implementation of Proposition 56 in California in 2017.

**Methods:**

Seven waves of Tobacco Use Supplements to the Current Population Survey (TUS-CPS) 2011–2019 data were used to obtain state-level aggregate self-reported outcomes, including cigarette price per pack, current and daily cigarette use, cigarette consumption per day, and current and daily use of non-cigarette tobacco products (hookah, pipe, cigar, and smokeless tobacco). A modified version of a synthetic control method was used to create a “synthetic” California that best resembled pre-policy sociodemographic characteristics and outcome trends in California while correcting time-invariant pre-policy differences. Various sensitivity analyses were also conducted.

**Results:**

The implementation of Proposition 56 was associated with an increase in self-reported cigarette price per pack in California ($1.844, 95%CI: $0.153, $3.534; p = 0.032). No evidence suggested that Proposition 56 was associated with the changes in the prevalence of current or daily cigarette use, cigarette consumption per day, or the prevalence of current or daily use of non-cigarette tobacco products.

**Conclusion:**

Most of the cigarette tax increase following Proposition 56 in California was passed on to consumers. There is a lack of evidence that the implementation of Proposition 56 was associated with the changes in the use of cigarettes and other tobacco products such as hookah, pipe, cigar, and smokeless tobacco.

## Introduction

While the overall tobacco use among adults has been declining in the U.S. [1], the use of cigarettes and non-cigarette tobacco products remains a public health concern. More than 480,000 deaths per year can be still attributed to cigarette smoking and secondhand smoke exposure [2]. The prevalence of tobacco use is increasing in some age and racial/ethnic subgroups [1, 3, 4].

Tax measures have generally been found to be one of the most effective strategies to prevent and reduce cigarette smoking among adults [5–7]. From 2015 to 2020 alone, a total of 21 states and Washington DC increased taxes on cigarettes [8]. There is little evidence suggesting that the remaining smokers are more nicotine dependent or price inelastic than those who have quit [9, 10]. However, as smoking prevalence becomes very low, a larger tax increase will be needed to retain the same percentage point reduction in smoking prevalence even if the price elasticity is not changed.

It is also suggested that the demand for non-cigarette tobacco products is responsive to price changes: the demand would be decreased in response to increases in their own prices but increased in response to increases in cigarette prices [11, 12]. Since 2015, a total of 28 states and Washington DC increased taxes on some tobacco products other than cigarettes [8]. Nevertheless, the effectiveness of recent tax measures on reducing non-cigarette tobacco use remains understudied. Particularly, whether substitutions between cigarettes and non-cigarette tobacco products would still happen when both taxes are increased by the same magnitude is uncertain.

In this study, we aim to evaluate the most recent tax measure in California, Proposition 56, which increased taxes on both cigarettes and non-cigarette tobacco products. After two narrowly failed previous attempts in 2006 and 2012, California voters approved the increase in tobacco excise taxes through Proposition 56 in November 2016. Effective in 2017, the cigarette tax in California increased by $2 from $0.87 to $2.87, with equivalent increases on other tobacco products. In 2016 prior to Proposition 56, the prevalence of overall tobacco use was 16.4% and the prevalence of cigarette smoking was 11.4% in California, both of which were the second-lowest in the U.S. [13].

Very few studies have provided empirical evidence on the effects of Proposition 56 on tobacco use. Keeler et al. found a decline in cigarette smoking prevalence among all Californians aged 21 years and older from 10.4% to 8.4% after Proposition 56 [14]. However, without a comparison group the decline in California during this period may simply reflect the nationwide downward trend in cigarette use. Another study by Boettiger et al. used synthetic control method and estimated that a 16.6% reduction in cigarette sales was seen following the Tobacco 21 law that increased the minimum legal sale age for tobacco products to 21 years (effective in 2016) and Proposition 56 in California [15]. Nonetheless, a decline in legal sales may not necessarily translate to a reduction in cigarette smoking prevalence because consumers can change their product purchasing patterns to mitigate price increases [16–19].

Our study adds to the existing literature in the following ways. First, we used difference-in-differences model in which the control group was created using synthetic control method to account for the contemporaneous trends in tobacco use and other confounding factors affecting tobacco use. Second, in addition to cigarette use, we also examined the changes in the use of non-cigarette tobacco products including hookah, pipe, cigar, and smokeless tobacco. Third, our data allowed us to focus on individuals 21 years old or above, so that we could remove the confounding from Tobacco 21 law that was implemented almost at the same time as Proposition 56 in California. Finally, the use of self-reported behavioral data can mitigate concerns about legal sales data.

## Methods

### Data source

We obtained self-reported tobacco use data from Tobacco Use Supplements to the Current Population Survey (TUS-CPS). Administered approximately every 3 to 4 years since 1992 as part of the U.S. Census Bureau’s Current Population Survey, TUS-CPS is a nationally representative survey that collects information on cigarette smoking and other tobacco use behaviors and perceptions. Each monthly wave surveyed approximately 240,000 civilian non-institutionalized adults aged 18 or older, about 8% of whom were sampled from California.

Our study period was 2011–2019. It was chosen to maintain comparability with previous studies [14, 15] and to keep a sufficiently large donor pool of control states for the construction of synthetic control given that states that implemented tobacco tax increases in the study period needed to be excluded to avoid bias in the estimates. There are seven waves of TUS-CPS in this period: January 2011, July 2014, January 2015, May 2015, July 2018, January 2019, and May 2019. We restricted our analysis to respondents aged 21 or older to avoid potential confounding from California’s Tobacco 21 law that went into effect in 2016.

### Outcome measures

Outcomes of interest were self-reported tobacco use measures aggregated at the state-level, including average cigarette price per pack ($2019), prevalence of current cigarette use (%), prevalence of daily cigarette use (%), average cigarette consumption per day among current cigarette users (number of cigarettes), prevalence of current hookah, cigar, pipe, and smokeless tobacco use (%), and prevalence of daily hookah, cigar, pipe, and smokeless tobacco use (%).

Cigarette price per pack was calculated as the average price paid for the last pack of cigarettes among current cigarette users. Current cigarette use was identified if a respondent reported having smoked at least 100 cigarettes in their lifetime and currently smoking cigarettes some days or every day. Daily cigarette use was identified if a current cigarette user currently smoked cigarettes every day. The following calculation was applied to average cigarette consumption per day among current users: for daily users, we used the reported number of cigarettes that they smoked each day on average; for some day cigarette users, we calculated their average cigarettes per day by multiplying the average number of cigarettes smoked on the days smoked by the fraction of the past 30 days the respondent smoked. Similar to the definition of current/daily cigarette use, we identified current/daily use of hookah, cigar, pipe, and smokeless tobacco.

### Predictor variables in the construction of synthetic control

We utilized the monthly Current Population Survey administered in the same wave as TUS-CPS to construct aggregate state-level sociodemographic characteristics. These time-varying predictors included unemployment rate (%), proportion of population aged 20 or below (%), proportion of Hispanics (%), proportion of non-Hispanic Blacks (%), proportion of non-Hispanic other minorities (%), and proportion of population with a high school diploma or less (%). To further increase the similarity between California and its synthetic control, we also included the presence of comprehensive smoke-free laws, minimum legal sales age for cigarettes > 18 laws, and recreational marijuana laws as predictors. These laws were coded as time-varying binary indicators, with the value of one if the laws were present during the survey wave in the state and zero otherwise. Cigarette sales per capita were constructed as the number of packs in a given state for which state excise taxes were paid divided by the state population size. The information on comprehensive smoke-free laws, minimum legal sales age for cigarettes laws, and cigarette sales was obtained from the Centers for Disease Control and Prevention State Tobacco Activities Tracking and Evaluation System. The information on recreational marijuana laws was obtained from the National Conference of State Legislatures. Since using all waves of the pre-policy outcomes would render all other predictors irrelevant [20], we only added two waves of outcomes prior to Proposition 56 as predictors (January 2011 and May 2015). Our results were not sensitive to how many waves were selected (two or three waves) or which waves were selected.

### Statistical analysis

To identify the changes in tobacco price and use following Proposition 56 in California, a control group is needed that can be used as a counterfactual (i.e., what would have occurred in California in the absence of Proposition 56). One common approach is to use the rest of the U.S. states to obtain counterfactual trends. However, since the rest of the U.S. states is likely to have different pre-policy sociodemographic characteristics and outcome trends compared to California, arguably a better approach is to create a “synthetic” control group based on the works by Abadie et al. [21, 22]. Specifically, in this study we constructed the synthetic California as a weighted combination of the donor pool—the 30 states that did not increase tobacco taxes in the study period. The remaining 19 states were not included in the donor pool because they increased tobacco taxes during the study period. These 19 states included Alabama, Connecticut, Delaware, Hawaii, Illinois, Kansas, Kentucky, Louisiana, Massachusetts, Minnesota, Nevada, New Hampshire, Pennsylvania, Rhode Island, Ohio, Oklahoma, Oregon, Vermont, and West Virginia. The weights were derived to minimize the mean squared differences in the sociodemographic characteristics and outcome trends between the actual and synthetic California before Proposition 56 went into effect in 2017. Following Abadie et al. [23], we restricted these weights to be in the [0, 1] interval and the sum of weights to be one. The synthetic California was constructed separately for each of the outcomes using all predictor variables described above.

If the pre-policy period is long enough and sociodemographic characteristics and outcome trends of the intervention state (California) can be reasonably fitted by its synthetic control, the difference in the outcomes between the intervention state and its synthetic control after policy implementation can be interpreted as the effects of the policy [21]. Nonetheless, if pre-policy fit is suboptimal and the differences in pre-policy trends between the intervention state and its synthetic control did not vary with time, Kreif et al. [24] showed that a difference-in-differences adjustment can be used to correct the estimates. We therefore followed Kreif et al. and other recent works to combine the synthetic control method and the traditional difference-in-differences method to remove such pre-policy time-invariant differences [24–27]. Specifically, we calculated the difference between the change in the average value of an outcome before and after the implementation of Proposition 56 in California and the corresponding change in the synthetic California.

Because large sample inferential techniques are not appropriate when the number of units in the control group is small [21, 22], we followed Abadie et al. [21, 22] to conduct permutation-based tests. Specifically, we first treated each of the 30 states in the donor pool as the intervention state in lieu of California, constructed the corresponding synthetic control, and computed the difference-in-differences estimate to adjust for pre-policy time-invariant differences. If the estimate in California was larger in magnitude relative to the estimates in these other states, we considered that the estimate in California was unlikely to have occurred by chance. The rank of the magnitude of the estimate in California relative to the magnitude of the estimates in these other states served as a permutation-based test p-value. We also reported the implied 95% confidence intervals from the permutation-based test p-value using the procedure outlined in Altman et al. [28].

Following the main analysis, three sensitivity checks were performed. First, we excluded neighboring states from the donor pool to avoid potential spillover effects. Since Nevada and Oregon were already excluded from the donor pool because they implemented tobacco tax increases in the study period, only Arizona was excluded in this sensitivity analysis. Second, following Boettiger et al. [15], we excluded New York from the donor pool since it implemented several important tobacco control measures during the study period. Third, we calculated alternative p-values based on Abadie et al. [21]. Specifically, after constructing a synthetic control for each of the control states in the donor pool, we compared post- to pre-policy root mean square prediction error (RMSPE) ratio in California to the corresponding ratios in the control states in the donor pool. An alternative p-value was then calculated as the proportion of post- to pre-policy RMSPE ratios in the donor pool that were at least as extreme as the ratio in California.

## Results

### Descriptive statistics of outcome variables in California without adjustments

**Fig 1:**
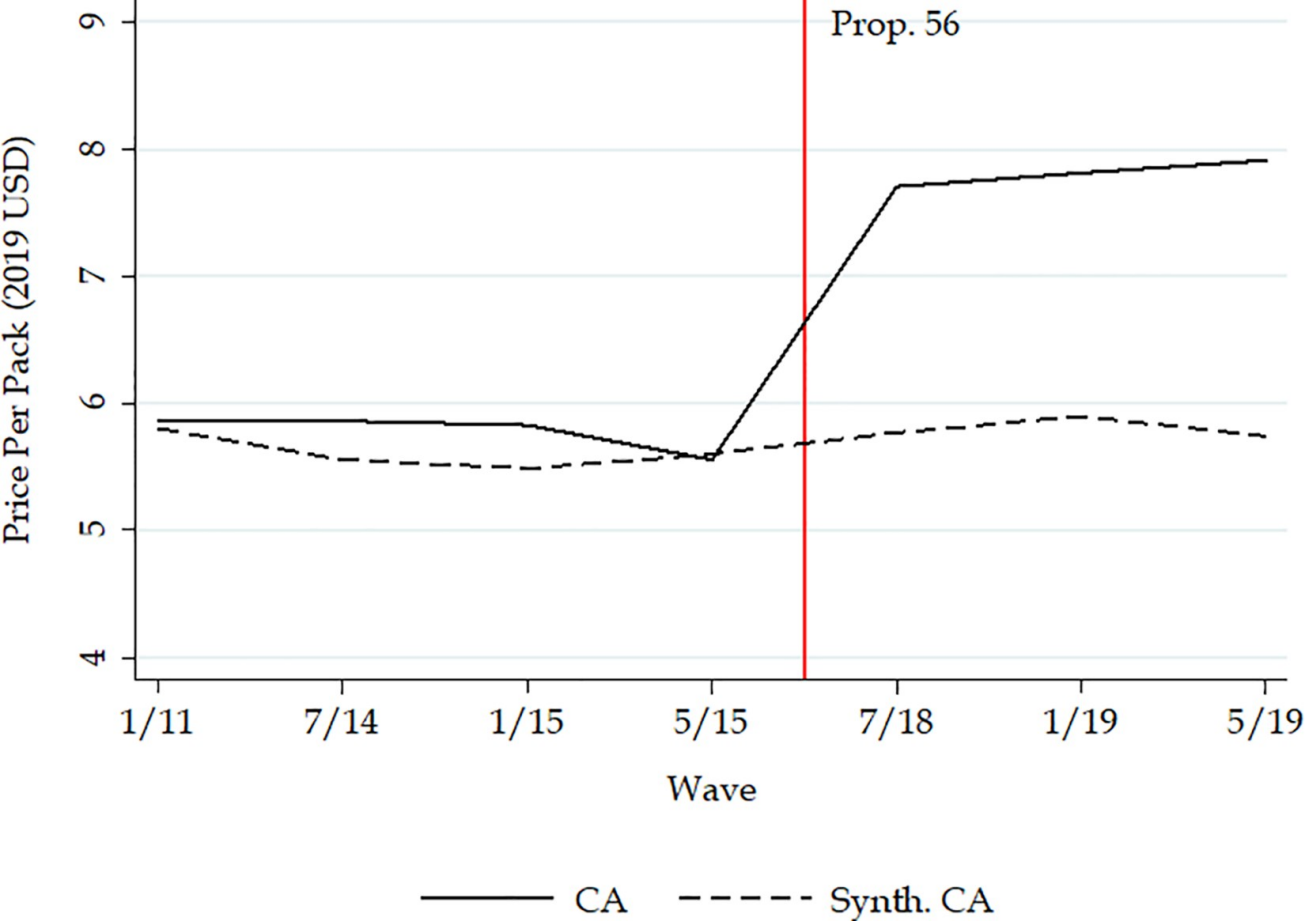
Comparison before and after the implementation of California Proposition 56 tobacco tax increase between California and synthetic control: Cigarette price ($2019).

The solid line in Fig 1 shows the unadjusted trend of cigarette price in California. From 2011 to 2015, the cigarette price per pack in California had been stable at around $6. In 2018, however, the price increased sharply to about $8 and was sustained at that level.

**Fig 2:**
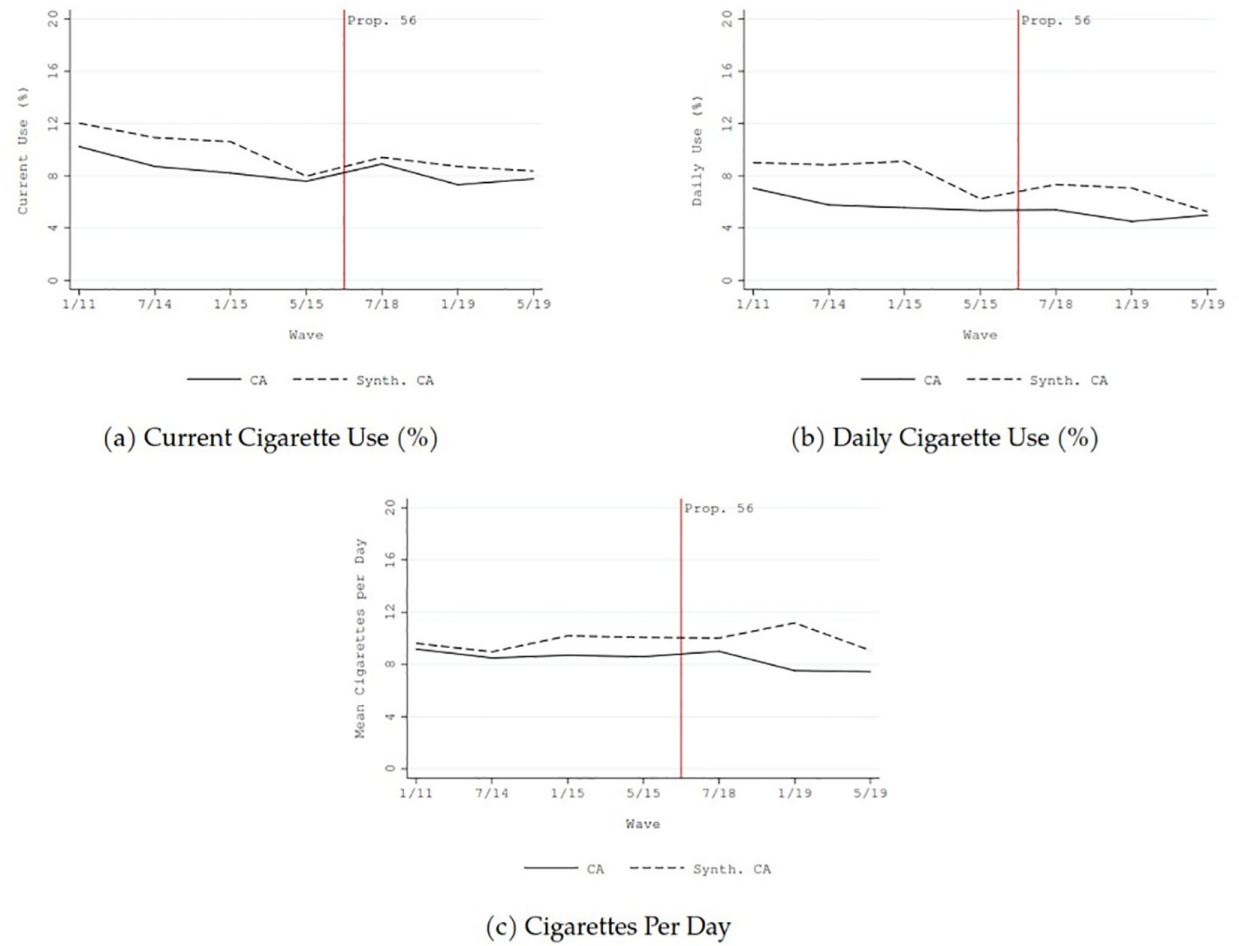
Comparison before and after the implementation of California Proposition 56 tobacco tax increase between California and synthetic control: Cigarette use outcomes.

**Fig 3:**
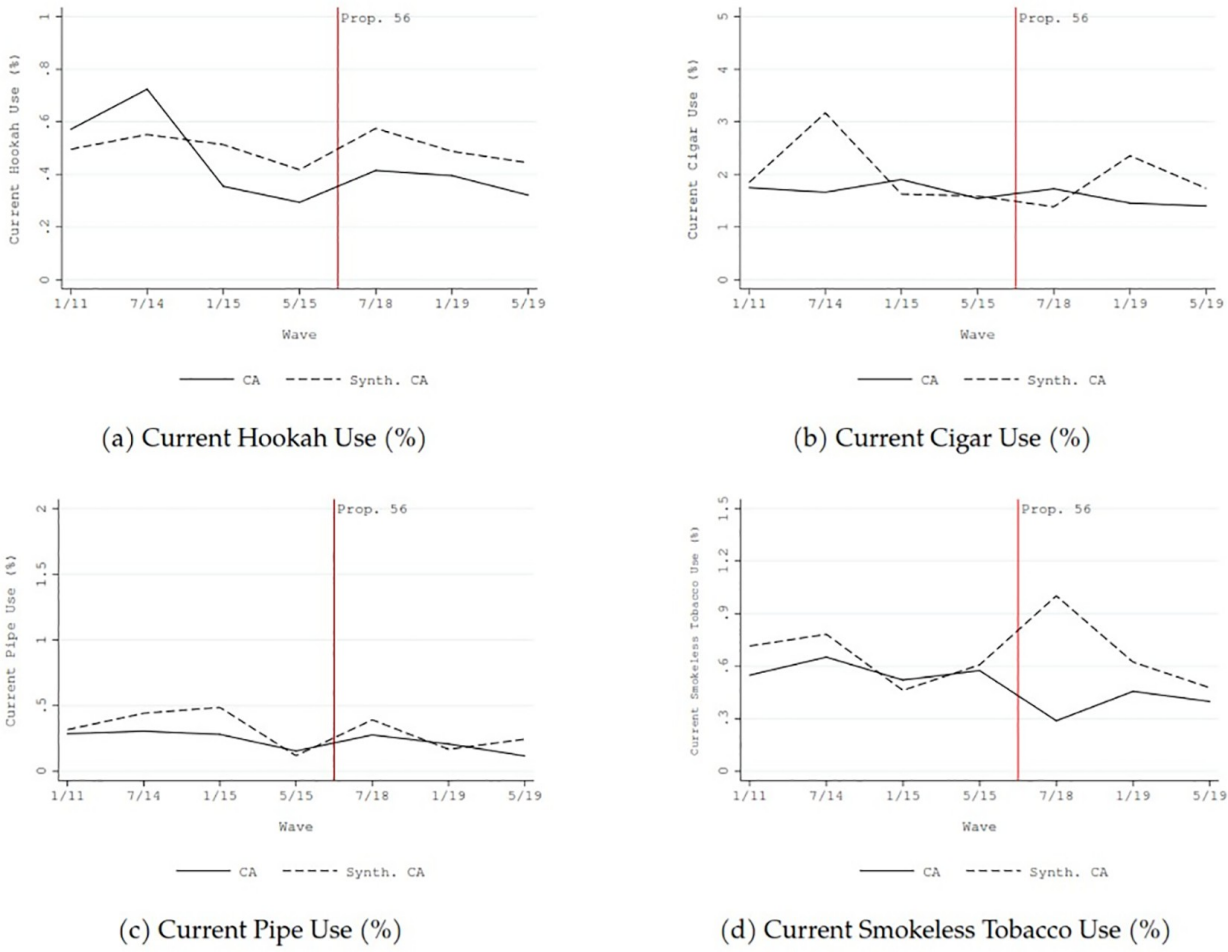
Comparison before and after the implementation of California Proposition 56 tobacco tax increase between California and synthetic control: Non-cigarette tobacco use outcomes—current use (%).

**Fig 4:**
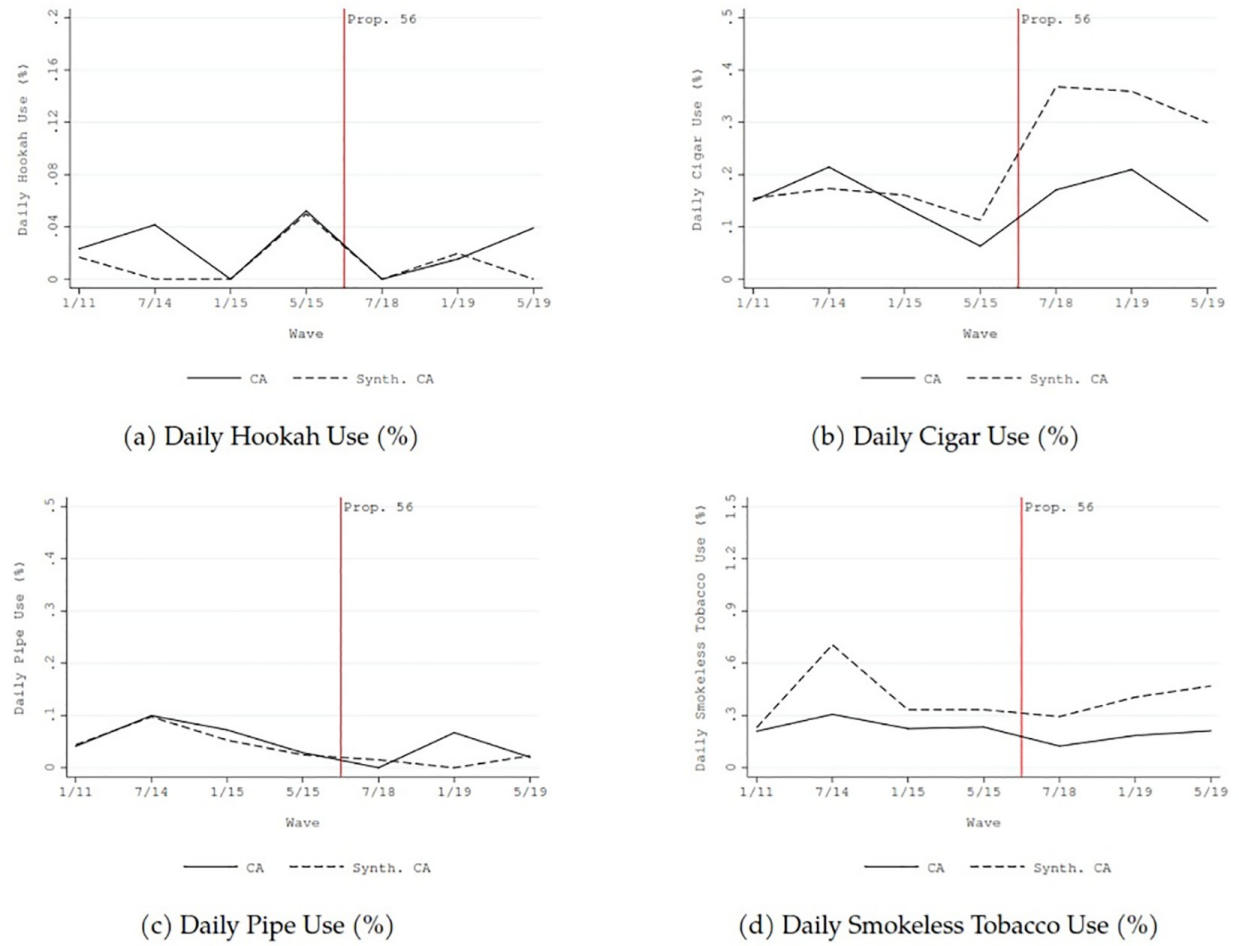
Comparison before and after the implementation of California Proposition 56 tobacco tax increase between California and synthetic control: Non-cigarette tobacco use outcomes—daily use (%).

From 2011 to 2019, the prevalence of current and daily cigarette use in California declined by approximately two percentage points, and the average number of cigarette consumed per day in California declined by about two (solid lines in Fig 2). Similar downward trends were also generally found for current use and daily use of non-cigarette tobacco in California (solid lines in Figs 3 and 4).

### Identification of synthetic control

The balance of the predictor variables between California and its synthetic control is shown in Table 1 (for cigarette price and use outcomes) and Tables 2 and 3 (for non-cigarette tobacco use outcomes). While some pre-policy sociodemographic characteristics were not closely matched, such as the proportion of non-Hispanic other minorities and cigarette sales per capita, the constructed synthetic California in general approximated the pre-policy sociodemographic characteristics and outcome trends in California. The weights used to create the synthetic control for each of the outcomes are reported in S1 and S2 Tables. For each outcome, New Mexico, Arizona, Colorado, Florida, and Utah often received sizable positive weights in the construction of synthetic California.

**Table 1:** Descriptive statistics of predictor variables in California and synthetic control: Cigarette price and use outcomes.

	Cigarette Price		Current Cigarette Use		Daily Cigarette Use		Cigarettes Consumption per Day among Current Cigarette Users	
	CA	Synth. CA	CA	Synth. CA	CA	Synth. CA	CA	Synth. CA
Outcome (1/11 Wave)	$5.86	$5.80	10.24%	12.03%	7.05%	9.01%	9.17	9.62
Outcome (5/15 Wave)	$5.55	$5.60	7.58%	7.97%	5.36%	6.24%	8.59	10.07
Unemployment Rate (%)	8.51	7.61	8.51	6.10	8.51	6.36	8.51	7.29
Proportion of Age<21 (%)	30.03	29.22	30.03	33.67	30.03	33.73	30.03	30.46
Proportion of Hispanics (%)	38.06	35.12	38.06	19.54	38.06	24.36	38.06	38.02
Proportion of Non-Hispanic Blacks (%)	5.49	7.87	5.49	5.63	5.49	1.42	5.49	2.10
Proportion of Non-Hispanic Other Minorities (%)	16.57	10.60	16.57	8.11	16.57	9.30	16.57	12.08
Proportion of Highest Education < = High School (%)	54.71	56.98	54.71	55.33	54.71	56.25	54.71	55.91
Comprehensive Smoke-free Laws	0.00	0.61	0.00	1.00	0.00	1.00	0.00	1.00
Minimum Legal Sales Age for Cigarettes > 18	0.00	0.00	0.00	0.61	0.00	0.57	0.00	0.05
Cigarette Sales per Capita (# of Packs)	23.33	35.17	23.33	23.43	23.33	22.29	23.33	28.27
Recreational Marijuana Legalization	0.00	0.00	0.00	0.01	0.00	0.06	0.00	0.17
RMSPE	0.232		1.872		2.577		1.099	

Notes. All variables were averaged at state-wave level for the 2011–2015 pre-policy period. The Root Mean Squared Prediction Error (RMSPE) is a measure of lack of fit between California and its synthetic control.

**Table 2:** Descriptive statistics of predictor variables in California and synthetic control: Non-cigarette use outcomes–current use.

	Hookah		Cigar		Pipe		Smokeless Tobacco	
	CA	Synth. CA	CA	Synth. CA	CA	Synth. CA	CA	Synth. CA
Outcome (1/11 Wave)	0.57	0.50	1.75	1.85	0.29	0.32	0.55	0.72
Outcome (5/15 Wave)	0.29	0.42	1.54	1.59	0.16	0.12	0.58	0.61
Unemployment Rate (%)	8.51	7.37	8.51	7.04	8.51	6.52	8.51	7.71
Proportion of Age<21 (%)	30.03	29.73	30.03	30.18	30.03	31.75	30.03	29.40
Proportion of Hispanics (%)	38.06	23.40	38.06	32.50	38.06	32.45	38.06	29.76
Proportion of Non-Hispanic Blacks (%)	5.49	7.51	5.49	6.90	5.49	7.48	5.49	7.73
Proportion of Non-Hispanic Other Minorities (%)	16.57	9.77	16.57	13.30	16.57	10.77	16.57	9.55
Proportion of Highest Education < = High School (%)	54.71	55.94	54.71	55.99	54.71	58.06	54.71	55.37
Comprehensive Smoke-free Laws	0.00	1.00	0.00	0.65	0.00	0.34	0.00	0.98
Minimum Legal Sales Age for Cigarettes > 18	0.00	0.09	0.00	0.05	0.00	0.16	0.00	0.44
Cigarette Sales per Capita (# of Packs)	23.33	29.59	23.33	38.86	23.33	31.58	23.33	26.98
Recreational Marijuana Legalization	0.00	0.00	0.00	0.00	0.00	0.04	0.00	0.00
RMSPE	0.138		0.767		0.125		0.111	

Notes. All variables were averaged at state-wave level for the 2011–2015 pre-policy period. The Root Mean Squared Prediction Error (RMSPE) is a measure of lack of fit between California and its synthetic control.

**Table 3:** Descriptive statistics of predictor variables in California and synthetic control: Non-cigarette use outcomes–daily use.

	Hookah		Cigar		Pipe		Smokeless Tobacco	
	CA	Synth. CA	CA	Synth. CA	CA	Synth. CA	CA	Synth. CA
Outcome (1/11 Wave)	0.02	0.02	0.15	0.15	0.04	0.04	0.21	0.23
Outcome (5/15 Wave)	0.05	0.05	0.06	0.11	0.03	0.02	0.23	0.33
Unemployment Rate (%)	8.51	6.85	8.51	7.44	8.51	7.39	8.51	7.47
Proportion of Age<21 (%)	30.03	28.18	30.03	29.86	30.03	30.01	30.03	29.96
Proportion of Hispanics (%)	38.06	28.75	38.06	25.64	38.06	28.94	38.06	32.37
Proportion of Non-Hispanic Blacks (%)	5.49	9.05	5.49	9.32	5.49	6.36	5.49	7.44
Proportion of Non-Hispanic Other Minorities (%)	16.57	7.65	16.57	9.59	16.57	10.31	16.57	8.13
Proportion of Highest Education < = High School (%)	54.71	55.51	54.71	55.91	54.71	56.07	54.71	55.72
Comprehensive Smoke-free Laws	0.00	0.50	0.00	0.62	0.00	0.75	0.00	1.00
Minimum Legal Sales Age for Cigarettes > 18	0.00	0.00	0.00	0.03	0.00	0.14	0.00	0.08
Cigarette Sales per Capita (# of Packs)	23.33	38.51	23.33	39.53	23.33	29.10	23.33	25.28
Recreational Marijuana Legalization	0.00	0.02	0.00	0.15	0.00	0.02	0.00	0.00
RMSPE	0.021		0.034		0.010		0.213	

Notes. All variables were averaged at state-wave level for the 2011–2015 pre-policy period. The Root Mean Squared Prediction Error (RMSPE) is a measure of lack of fit between California and its synthetic control.

The dashed lines in Figs 1–4 show the counterfactual estimates of what would have occurred in California in the absence of Proposition 56: the cigarette price would have been much lower and tobacco use in general would have declined anyway after 2017.

### Difference-in-differences estimates

The difference-in-differences estimates on the associations between California Proposition 56 tobacco tax increase and tobacco outcome variables are reported in Table 4.

**Table 4:** Difference-in-differences estimates on the associations between California Proposition 56 tobacco tax increase and tobacco outcomes.

	Average Pre-Post Difference: CA^1^	Average Pre-Post Difference: Synthetic CA^2^	Difference-in-Difference Estimates^3^	P-Value, P(|Δ other| ≥ |Δ CA|)^4^	Implied 95% CI from the P-value^5^		Alternative P-Value Based on Post/Pre-Policy RMSPE Ratio^6^	Implied 95% CI from P-value (RMSPE Ratio)^7^	
					Lower Limit	Upper Limit		Lower Limit	Upper Limit
**Cigarette**									
Cigarette Price per Pack ($2019)	2.035	0.191	1.844	0.032	0.153	3.534	0.032	0.153	3.534
Current Cigarette Use (%)	-0.690	-1.554	0.864	0.581	-2.109	3.838	0.935	-18.291	20.020
Daily Cigarette Use (%)	-0.970	-1.745	0.775	0.484	-1.348	2.897	0.935	-16.392	17.941
Cigarette Consumption per Day among Current Cigarette Users (#)	-0.751	0.378	-1.128	0.129	-2.585	0.328	0.226	-2.946	0.690
**Hookah**									
Current Use (%)	-0.109	0.008	-0.117	0.742	-0.776	0.543	0.645	-0.594	0.361
Daily Use (%)	-0.011	-0.010	-0.001	0.968	-0.044	0.042	0.516	-0.004	0.002
**Pipe**									
Current Use (%)	-0.056	-0.073	0.017	0.839	-0.135	0.168	0.806	-0.110	0.143
Daily Use (%)	-0.031	-0.042	0.010	0.871	-0.107	0.128	0.129	-0.003	0.024
**Cigar**									
Current Use (%)	-0.187	-0.233	0.046	0.935	-0.971	1.063	0.806	-0.299	0.391
Daily Use (%)	0.023	0.192	-0.169	0.258	-0.461	0.122	0.065	-0.349	0.010
**Smokeless Tobacco**									
Current Use (%)	-0.193	0.059	-0.252	0.645	-1.285	0.781	0.065	-0.520	0.016
Daily Use (%)	-0.070	-0.011	-0.059	0.806	-0.501	0.383	0.710	-0.354	0.236

Notes.

1) Calculated as the change in the average value of the outcome before and after Proposition 56 in California.

2) Calculated as the change in the average value of the outcome before and after Proposition 56 in synthetic California.

3) Calculated as the difference between 1) and 2).

4) Calculated using the distribution of the difference-in-differences estimates for the 30 control states in the donor pool. Specifically, the p-value of the two-sided test was calculated as the proportion of difference-in-differences estimates that were at least as extreme in absolute value as the estimate in California.

5) Calculated using the p-value in 4) based on the procedure outlined in Altman and Bland (2011).

6) Calculated using the distribution of post- to pre-policy RMSPE ratios for the 30 control states in the donor pool. Specifically, the p-value was calculated as the proportion of RMSPE ratios that were at least as extreme as the RMSPE ratio in California.

7) Calculated using the p-value in 6) based on the procedure outlined in Altman and Bland (2011).

#### Cigarette price

The implementation of Proposition 56 was associated with a $1.844 increase in cigarette price per pack in California (95% CI: $0.153, $3.534; p = 0.032).

#### Cigarette use

We did not find evidence that the implementation of Proposition 56 was associated with the changes in current cigarette use, daily cigarette use, or cigarette consumption per day.

#### Non-cigarette tobacco use

Further, we did not find evidence that the implementation of Proposition 56 was associated with the changes in non-cigarette tobacco use outcomes (current and daily use of hookah, pipe, cigar, and smokeless tobacco).

#### Sensitivity checks

The estimated associations were robust to excluding California’s neighboring state Arizona (S3 Table) or New York (S4 Table). The alternative p-values are reported in the second last column of Table 4, which were qualitatively consistent with p-values reported in the main analysis (the 5^th^ column of Table 4).

## Discussion

Applying very similar methods but different data sources, our finding on the association between California Proposition 56 and cigarette price was comparable to Boettiger et al. [15] ($1.844 increase in self-reported price per pack in our study vs. $1.89 increase in sales price per pack in Boettiger et al. [15]). This finding suggested that most of the rise in cigarette excise tax was passed on to consumers (~90%). It is worth noting that tax burden is unlikely to be shared equally across groups. Future research is warranted to examine the financial burden caused by increased tobacco taxes, particularly among low-income groups [29].

We did not find evidence that California Proposition 56 was associated with the changes in cigarette use, in juxtaposition to Boettiger et al. [15] that found a reduction in legal sales of cigarettes after Proposition 56. There could be several explanations for these different results. First, if consumers bought a large volume of cigarettes in anticipation of the tax increase (i.e., stockpiling), cigarette use prevalence might not decline in the short term (as reported in our study) even though cigarette sales saw a reduction following Proposition 56 (as reported in Boettiger et al. [15]). In this case, a longer post-policy study period is required to observe a decline in cigarette use. Second, if more consumers purchased tobacco from alternative sources (such as illegal market or other states) or consumers bought a larger quantity from alternative sources following Proposition 56, legal sales in California may decline but self-reported use may not. Third, self-reported cigarette use outcomes may be subject to reporting bias. This explanation is probably implausible, however, because the finding on self-reported price was consistent with that on sales price in Boettiger et al. [15].

Despite the differences in point estimates on cigarette use between our study and Boettiger et al. [15], we would like to point out that the 95% confidence intervals associated with our estimates were wide enough to include the point estimate in Boettiger et al. [15]. For instance, the lower limit of the 95% confidence interval associated with cigarette consumption per day was -2.6, corresponding to a 30% decline relative to the value in California in the latest wave of TUS-CPS prior to Proposition 56. Boettiger et al. [15] estimated a 16.6% reduction in cigarette sales following Proposition 56, falling into the confidence interval estimated in our study. This implied that our study did not reject the findings in Boettiger et al. [15].

In addition to the estimates on cigarette price and use outcomes, we contributed to the existing literature by examining the associations between Proposition 56 and non-cigarette tobacco use outcomes. Corroborating the findings on cigarette use, we did not find Proposition 56 associated with the changes in hookah, pipe, cigar, or smokeless tobacco use. This implied that, when cigarette and non-cigarette tobacco excise taxes increased by similar levels, cigarettes and non-cigarette tobacco products were not substituted for each other. If substituting non-cigarette tobacco for cigarettes is desirable due to harm reduction benefits, higher taxes on cigarettes might be needed. Taxes on non-cigarette tobacco products have been understudied compared to the numerous research papers on cigarette taxes. Future research is needed to investigate the impacts of recent non-cigarette tobacco tax increases on the use of both cigarette and non-cigarette products in California as well as other jurisdictions in the world.

This study is not without limitations. First, even though we utilized synthetic control method coupled with difference-in-differences method, we were not able to control for the unobserved time-variant confounding factors such as social norms. Hence our findings should not be interpreted as causality. Some electronic cigarette products such as JUUL were introduced during the post-policy period, the availability of which might be disproportionately more in California compared to control states in the donor pool. Should there have been such disproportionate availability, it might confound the association estimates. Previously, electronic cigarettes have been found to be substitutes for cigarettes and non-cigarette tobacco products investigated in this study [30, 31]. We argue that such substitution would have contributed to a downward bias, yielding the lower bound of the estimated associations. Therefore, the lack of evidence that Proposition 56 in California was associated with a reduction in traditional tobacco use would still hold with the introduction of electronic cigarettes taken into consideration. Second, the state-level analysis is subject to ecological fallacy (i.e., incorrectly explaining an inference about an individual with aggregate data for a group) and should not be interpreted as individual-level associations. Third, although TUS-CPS is one of the best data sources one can utilize to study tobacco use in the U.S., the self-reporting bias may be still a concern. Fourth, the analysis was limited by data availability. For example, we were not able to observe the long-term changes in tobacco use behaviors after Proposition 56. We were not able to examine electronic cigarette use, which may have become common among adults in recent years. We were not able to examine non-cigarette tobacco prices or consumption, either. Further, the pre-policy fit between California and the synthetic California might be suboptimal, but we were unable to test the assumption of pre-policy parallel time trends because large sample inferential techniques are not appropriate when the number of units in the control group is small [21]. To address this concern, we applied difference-in-differences adjustment to account for time-invariant differences between California and the synthetic California. We also calculated alternative p-values taking into consideration of the deviation between California and the synthetic California in the post-policy period based on Abadie et al. [21]; these p-values were qualitatively similar to the p-values without this consideration. Lastly, the estimates in our study were averaged at the whole-population level. Recent research has noted that some segments of tobacco users (e.g., low-income group) are likely to be more price-sensitive than others [32, 33]. Whether California Proposition 56 has generated larger impacts on some subgroups remains unknown.

Notwithstanding the limitations, this study informs policymakers interested in implementing tobacco tax increase in places where the prevalence of tobacco use is already low. Following the recent success in reducing tobacco use globally [34], we believe that these findings are increasingly relevant. It should be noted, however, that our findings should not be interpreted as evidence that the demand for tobacco products does not respond to changes in its prices. Rather, it suggests that the effects of tax increases may not be as large as before when tobacco use prevalence is already very low [35]. In this case, policymakers may want to consider even higher tobacco taxes as well as comprehensive tobacco control policies to induce behavioral changes in the remaining tobacco users.

## Conclusion

Proposition 56 in California in the U.S. increased excise taxes on cigarettes and non-cigarette tobacco products by $2 per pack or equivalent. This study suggested that self-reported cigarette price was increased by $1.844 per pack following Proposition 56. It appears that most of the cigarette tax increase was passed on to consumers. However, there is a lack of evidence that the implementation of Proposition 56 was associated with the changes in the use of cigarettes and other tobacco products such as hookah, pipe, cigar, and smokeless tobacco.

## Supporting information

S1 Table Weights used to construct synthetic control for cigarette price and use outcomes. (DOCX)

S2 Table Weights used to construct synthetic control for non-cigarette use outcomes. (DOCX)

S3 Table Sensitivity analysis of difference-in-difference estimates: Excluding California neighboring states from synthetic control donor pool. (DOCX)

S4 Table Sensitivity analysis difference-in-difference estimates: Excluding New York from synthetic control donor pool. (DOCX)
