# Supplementary material for: Tobacco price and use following California Proposition 56 tobacco tax increase
Source: PLoS One. 2021 Oct 13;16(10):e0257553. doi: 10.1371/journal.pone.0257553 (PMC8513910; doi:10.1371/journal.pone.0257553)
Supplement: S1 Table — (DOCX) [file pone.0257553.s001.docx]

**S1 Table. Weights Used to Construct Synthetic Control for Cigarette Price and Use Outcomes**

| **Donor States** | **Weights** | | | |
| --- | --- | --- | --- | --- |
|  | **Cigarette Price** | **Current Cigarette Use** | **Daily Cigarette Use** | **Cigarette Consumption per Day among Current Cigarette Users** |
| Alaska | 0 | 0 | 0 | 0 |
| Arizona | 0 | 0 | 0 | 0 |
| Arkansas | 0 | 0 | 0 | 0 |
| Colorado | 0 | 0 | 0 | 0.229 |
| Florida | 0.282 | 0 | 0 | 0 |
| Georgia | 0.066 | 0 | 0 | 0 |
| Idaho | 0 | 0 | 0 | 0 |
| Indiana | 0 | 0 | 0 | 0 |
| Iowa | 0 | 0 | 0 | 0 |
| Maine | 0 | 0 | 0 | 0 |
| Maryland | 0 | 0.158 | 0 | 0 |
| Michigan | 0 | 0 | 0 | 0 |
| Mississippi | 0 | 0 | 0 | 0 |
| Missouri | 0.045 | 0 | 0 | 0 |
| Montana | 0 | 0 | 0 | 0 |
| Nebraska | 0 | 0 | 0 | 0 |
| New Jersey | 0 | 0 | 0 | 0 |
| New Mexico | 0.607 | 0.223 | 0.353 | 0.722 |
| New York | 0 | 0 | 0 | 0 |
| North Carolina | 0 | 0 | 0 | 0 |
| North Dakota | 0 | 0 | 0 | 0 |
| South Carolina | 0 | 0 | 0 | 0 |
| South Dakota | 0 | 0 | 0 | 0 |
| Tennessee | 0 | 0 | 0 | 0 |
| Texas | 0 | 0 | 0 | 0 |
| Utah | 0 | 0.606 | 0.573 | 0.049 |
| Virginia | 0 | 0 | 0 | 0 |
| Washington | 0 | 0.013 | 0.074 | 0 |
| Wisconsin | 0 | 0 | 0 | 0 |
| Wyoming | 0 | 0 | 0 | 0 |

Notes. States that increased tobacco excise taxes in the study period were excluded from the donor pool (Alabama, Connecticut, Delaware, Hawaii, Illinois, Kansas, Kentucky, Louisiana, Massachusetts, Minnesota, Nevada, New Hampshire, Pennsylvania, Rhode Island, Ohio, Oklahoma, Oregon, Vermont, and West Virginia).
