# Supplementary material for: Tobacco price and use following California Proposition 56 tobacco tax increase
Source: PLoS One. 2021 Oct 13;16(10):e0257553. doi: 10.1371/journal.pone.0257553 (PMC8513910; doi:10.1371/journal.pone.0257553)
Supplement: S3 Table — (DOCX) [file pone.0257553.s003.docx]

**S3 Table. Sensitivity Analysis of Difference-in-differences Estimates: Excluding California Neighboring States from Synthetic Control Donor Pool^1^**

|  | **Difference-in-Differences Estimates^2^** | **P-Value, P(\|Δ other\| ≥ \|Δ CA\|)^3^** | **Implied 95% CI from P-Value^4^** | | **Alternative P-Value Based on Post/Pre-Policy RMSPE Ratio^5^** | **Implied 95% CI from P-Value (RMSPE Ratio)^6^** | |
| --- | --- | --- | --- | --- | --- | --- | --- |
|  |  |  | **Lower Limit** | **Upper Limit** |  | **Lower Limit** | **Upper Limit** |
| **Cigarette** |  |  |  |  |  |  |  |
| Cigarette Price per Pack ($2019) | 1.852 | 0.033 | 0.143 | 3.561 | 0.033 | 0.143 | 3.561 |
| Current Cigarette Use (%) | 0.850 | 0.633 | -2.517 | 4.217 | 0.967 | -35.355 | 37.055 |
| Daily Cigarette Use (%) | 0.773 | 0.500 | -1.423 | 2.970 | 0.967 | -32.169 | 33.716 |
| Cigarette Consumption per Day among Current Cigarette Users | -1.110 | 0.200 | -2.802 | 0.582 | 0.233 | -2.926 | 0.707 |
|  |  |  |  |  |  |  |  |
| **Hookah** |  |  |  |  |  |  |  |
| Current Use (%) | -0.136 | 0.633 | -0.676 | 0.404 | 0.533 | -0.554 | 0.282 |
| Daily Use (%) | 0.001 | 0.933 | -0.031 | 0.034 | 0.533 | -0.003 | 0.006 |
|  |  |  |  |  |  |  |  |
| **Pipe** |  |  |  |  |  |  |  |
| Current Use (%) | 0.079 | 0.667 | -0.267 | 0.426 | 0.900 | -1.063 | 1.222 |
| Daily Use (%) | 0.011 | 0.900 | -0.152 | 0.175 | 0.300 | -0.010 | 0.033 |
|  |  |  |  |  |  |  |  |
| **Cigar** |  |  |  |  |  |  |  |
| Current Use (%) | 0.042 | 0.933 | -0.855 | 0.938 | 0.833 | -0.322 | 0.406 |
| Daily Use (%) | -0.119 | 0.533 | -0.483 | 0.245 | 0.133 | -0.274 | 0.036 |
|  |  |  |  |  |  |  |  |
| **Smokeless Tobacco** |  |  |  |  |  |  |  |
| Current Use (%) | -0.232 | 0.700 | -1.363 | 0.898 | 0.300 | -0.668 | 0.203 |
| Daily Use (%) | -0.154 | 0.600 | -0.711 | 0.403 | 0.233 | -0.406 | 0.098 |

Notes.

1. Since Nevada and Oregon were already excluded from the donor pool because they implemented tobacco tax increases in the study period, only Arizona was excluded in this sensitivity analysis.
2. Calculated as the difference between the change in the average value of the outcome before and after Prop. 56 in California and the corresponding change in synthetic California.
3. Calculated using the distribution of the difference-in-differences estimates for the 30 control states in the donor pool. Specifically, the p-value of the two-sided test was calculated as the proportion of difference-in-differences estimates that were at least as extreme in absolute value as the estimate in California.
4. Calculated using the p-value in 4) based on the procedure outlined in Altman and Bland (2011).
5. Calculated using the distribution of post- to pre-policy RMSPE ratios for the 30 control states in the donor pool. Specifically, the p-value was calculated as the proportion of RMSPE ratios that were at least as extreme as the RMSPE ratio in California.
6. Calculated using the p-value in 6) based on the procedure outlined in Altman and Bland (2011).
